# Potassium Permanganate-Impregnated Amorphous Silica–Alumina Derived from Sugar Cane Bagasse Ash as an Ethylene Scavenger for Extending Shelf Life of Mango Fruits

**DOI:** 10.1021/acsomega.3c08119

**Published:** 2024-02-02

**Authors:** Napassorn Chanka, Waleeporn Donphai, Metta Chareonpanich, Kajornsak Faungnawakij, Günther Rupprechter, Anusorn Seubsai

**Affiliations:** † Department of Chemical Engineering, Faculty of Engineering, Kasetsart University, Bangkok 10900, Thailand; ‡ Center of Excellence on Petrochemical and Materials Technology, Kasetsart University, Bangkok 10900, Thailand; § National Nanotechnology Center (NANOTEC), National Science and Technology Development Agency (NSTDA), Pathum, Thani 12120, Thailand; ∥ Institute of Materials Chemistry, Technische Universität Wien, Getreidemarkt 9/BC, Vienna 1060, Austria

## Abstract

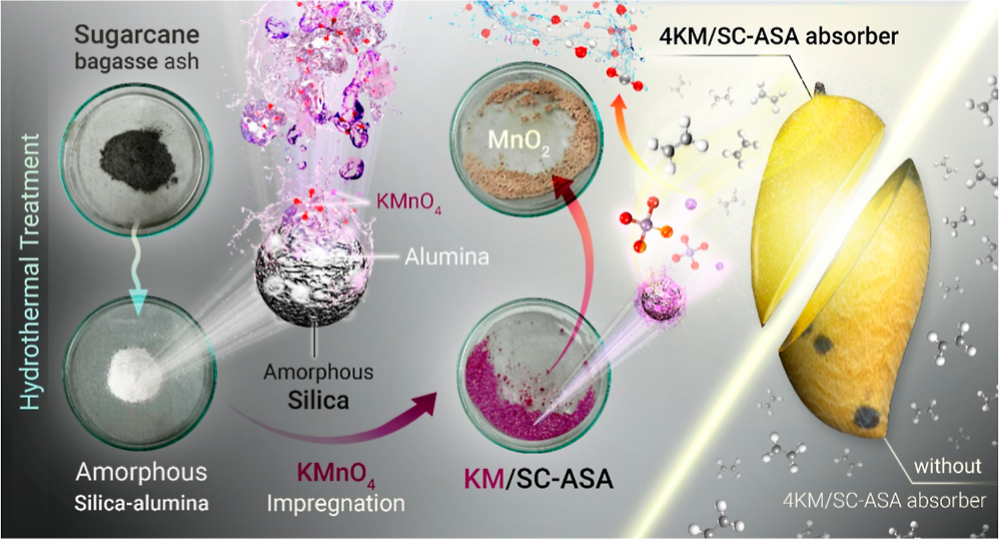

Ethylene, a plant hormone, is a gas that plays a crucial role in fruit ripening and senescence. In this work, a novel ethylene scavenger was prepared from amorphous silica–alumina derived from sugar cane bagasse ash (SC-ASA) and used to prolong the shelf life of mango fruits during storage. KMnO_4_ at 2, 4, or 6 wt %/w was loaded on SC-ASA using an impregnation method. The results showed that 4% w/w KMnO_4_ loaded on SC-ASA (4KM/SC-ASA) was superior for ethylene removal at an initial ethylene concentration of 400 μL L^–1^ for 120 min under ambient conditions (25–27 °C and 70–75% relative humidity), resulting in 100% ethylene removal. The kinetic study of ethylene removal showed that the adsorption data were best fitted with a pseudo-first-order kinetic model. The effects of 4KM/SC-ASA as sachets on the quality changes of the mango fruits were investigated, with the results showing that mango fruits packed in cardboard boxes with 4KM/SC-ASA had significantly delayed ripening, low levels of ethylene production, respiration, and weight loss, high fruit firmness, low total soluble solids, and high acidity compared to those of the control treatment. These findings should contribute to developing an ethylene scavenger to extend the shelf life of fruits, reduce the waste of the sugar and ethanol industries, and make it a valuable material.

## 1 Introduction

Mango (*Mangifera indica L.*) is one of the world’s most popular fruits. Mango is widely cultivated in tropical and subtropical regions, led by India, China, Thailand, Mexico, Pakistan, and Indonesia.^1,2^ In Thailand, the main mango cultivars include Nam Dok Mai, Khiew Sawoey, and Ok Rong, which occupy an area of 910 million ha with a production of 900 million tons in 2021.^3^ Mango has unique characteristics, such as a favorable flavor and aroma, high levels of antioxidants, as well as being a rich source of the nutrients required for a healthy life and reduced risk of fatal diseases.^4,5^ Mango is classified as a climacteric fruit, which can generate ethylene (C_2_H_4_) by itself. It involves a sequence of biological, physiological, and structural controlled changes that cause color, flavor, texture, and taste alterations.^6,7^ Generally, C_2_H_4_ production and respiration occur within the fruit during ripening and affect a series of metabolic activities, such as hydrolysis of starch into sugar, leading to an increase in total soluble solid (TSS) contents and a decrease in acidity, changes in structural polysaccharides leading to flesh softening, chlorophyll degradation and carotenoid biosynthesis, changes in fruit color, and biosynthesis of volatile compounds.^8,9^

Moreover, the storage of mangoes is recommended at temperatures of 13 to 15 °C and % relative humidity (% RH) of 85 to 95% to delay fruit physiological deterioration, maintain quality, and prolong postharvest life.^10^ Velasquez et al. (2023) exhibited that mango fruits shipped at low temperatures (8 °C) have poor consumer acceptance because of low dry matter, chilling injury (CI) disorders, and other storage disorders, resulting in grayish, scaldlike skin discoloration, electrical spotting, skin pitting, loss of flesh color, and flesh browning. Moreover, they suggested that the mango fruits should be transported at 10–12.5 °C to avoid chilling damage during transportation and ensure high quality.^10^ Cantre et al. (2017) showed that CI symptoms appear after the product has been removed from cold storage. The development of CI in mango fruits significantly affected tissue structure and pore networks by causing drastic changes in tissue aeration and disrupting the normal respiratory metabolism, which is associated with cell leakage, a decrease in pore size, and an increase in pore fragmentation and pore-specific area.^11^ However, throughout most of the country, Thailand has tropical and humid conditions all year round, so mango is commonly transported by refrigerated trucks and stored in the refrigerator. Moreover, low-temperature storage (<10 °C) can cause cold storage damage or CI, including uneven fruit ripening, typical skin symptoms, and flesh damage, resulting in poor satisfactory quality for consumers.^10^ In addition, unripe mangoes should be stored at room temperature because the mango fruits would continue to ripen, growing sweeter and softer. Therefore, excessive ethylene in the packaging environment at room temperature must be removed to reduce shipping costs and preserve fresh produce during shipping, storage, and handling.

Nowadays, one of the most common ethylene-scavenging technologies used in active packaging is based on potassium permanganate (KMnO_4_). KMnO_4_ is an inorganic chemical compound that acts as a strong oxidizing agent in food packaging applications. KMnO_4_ adsorbs ethylene from the atmosphere around horticultural products into carbon dioxide (CO_2_), water (H_2_O), and manganese(IV) dioxide (MnO_2_). It changes color from the purple permanganate ion (MnO_4_^–^) to the brown MnO_2_ ion.^12^ Several studies have confirmed that the application of KMnO_4_ can reduce exogenous C_2_H_4_ and delay the ripening and senescence of climacteric fruits, such as banana, mango, kiwifruit, avocado, and apple.^13−15^ Mansourbahmani et al. (2016) investigated various ethylene-scavenging treatments on tomatoes in a cold room at 7 ± 0.5 °C and 90 ± 2% relative humidity. The order of ethylene removal performance in the different treatments was palladium-promoted nanozeolite > KMnO_4_ > 1-MCP > SA = CaCl_2_ > UV-C.^16^ However, KMnO_4_ is more suitable than palladium because it is more economical.

Since KMnO_4_ should not be applied directly on postharvested products because of its toxicity and purple color stain,^12,17,18^ it is used practically as a sachet in packaged fruit, with natural convection and diffusion being the only driving forces for the reaction between the C_2_H_4_ molecules and the oxidant in the atmosphere.^13,14^ Thus, inert porous materials, such as activated carbon.,^19^ alumina beads,^14^ alumina nanoparticle-incorporated-carbon nanofibers,^13^ clay,^20^ and zeolite,^21^ should be used as a support for KMnO_4_. There has been extensive research on using KMnO_4_-based materials for ethylene removal. For example, Álvarez-Hernández et al. (2019) used KMnO_4_ on sepiolite to maintain the quality and delay the ripening of apricots.^22^ Wills et al. (2004) investigated KMnO_4_-alumina absorbent at 20 °C and 90% RH to remove low levels of ethylene from the atmosphere.^14^ Sammi and Masud (2009) examined how using KMnO_4_ contributed to producing CO_2_ and H_2_O in tomato packaging, decreasing respiration rates and speeding up the ripening processes.^23^ Tirgar et al. (2018) produced KMnO_4_ loaded into the high surface area of alumina nanofiber membranes, leading to increased ethylene-scavenging performance.^13^ In addition, there are several commercial C_2_H_4_ scavengers in the form of highly permeable sachets containing KMnO_4_ immobilized on a porous support material such as Ethylene Control (Ethylene Control Inc.; USA), GreenPack (Rengo Co. Ltd.; Japan), Ethylene EliminatorPak (Desiccare Inc.; USA), Everfresh and EthylSachet (ECP Ltd.; USA), BI-ON SORB (Bioconservacion S.A.; Spain), BEfresh (Alpine Foods Co., Ltd.; Thailand), Ethyl Stopper (ProFresh Systems Pty Ltd.; Australia), and SofnofilTM (Molecular Products Ltd.; UK).

In Thailand, large amounts of sugar cane bagasse ash (SC) from sugar processing sites are currently used in the sugar and bioethanol manufacturing processes, creating plenty of solid waste ash.^24^ These ash wastes are not valuable and are generally disposed of on agricultural land. Thus, the utilization of SC would have a positive effect from both economic and ecological points of view. Typically, SC contains an abundant silica (SiO_2_) content (above 60%).^25^ Therefore, SC could be used as an alternative source of SiO_2_ to generate low-cost and environmentally friendly materials.^26^

Amorphous silica–alumina (ASA) is commonly used as an active catalyst or as a functional support for active groups. ASA materials represent an important class of porous inorganic solids with relative advantages, such as a high surface area with a large number of acid sites and a wide distribution of pore sizes in micro- and mesoporous regions.^27^ Suganuma et al. (2020) analyzed the adsorption kinetics of nitrogen (N_2_)-containing compounds on ASA and reported that the adsorption rate was pseudo-first-order, indicating that the adsorption rate was controlled by the diffusion of compounds in the pores of ASA.^28^ Hence, ASA is expected to have high adsorption capacities.

To the best of our knowledge, none of the published research studies has explored the development and characterization of ASA from SC-based C_2_H_4_ adsorbents to extend the shelf life of fresh fruit. In this work, we synthesized KMnO_4_-impregnated ASA derived from SC at different KMnO_4_ loadings. Therefore, the synthesized adsorbents as a C_2_H_4_ scavenger to monitor the quality attributes of mango (*M. indica L.*) fruits can be used in cardboard boxes and stored under ambient conditions. Furthermore, the physical and chemical parameters of the adsorbent (such as the phase component, porosity, surface area, shape, and elemental composition) and the developed adsorption kinetic model were also investigated in order to determine how these relate to their ethylene adsorption performance.

## 2 Results and Discussion

### 2.1 KMnO_4_ Impregnated with Amorphous Silica–Alumina from SC

The chemical components of the SC and SC-derived amorphous silica–alumina (SC-ASA) were determined using X-ray fluorescence spectrometry (XRF), as shown in Table 1. The SC mainly consisted of SiO_2_ (64.90%) with other small components (each <10%), such as K_2_O, CaO, Al_2_O_3_, and Fe_2_O_3_. These values indicated the high content of SiO_2_ in SC, which has potential as a low-cost silicon source for SC-ASA synthesis.^29^ After hydrothermal treatment, the chemical components of the synthesized SC-ASA were Na_2_O (32.90%), SiO_2_ (31.90%), and Al_2_O_3_ (29.60%), roughly equal to a Si-to-Al molar ratio of 0.86.

**Table 1:** Chemical Compositions of SC and SC-ASA^a^

	% weight	
chemical composition	SC	SC-ASA
SiO_2_	64.88	33.20
Al_2_O_3_	5.680	32.52
Na_2_O	N/A	23.01
K_2_O	9.910	2.770
CaO	6.450	0.030
Fe_2_O_3_	3.490	0.040
ETC	9.590	8.430

a N/A = not applicable.

### 2.2 Material Characterization

#### 2.2.1 X-ray Diffraction Analysis

**Figure 1:**
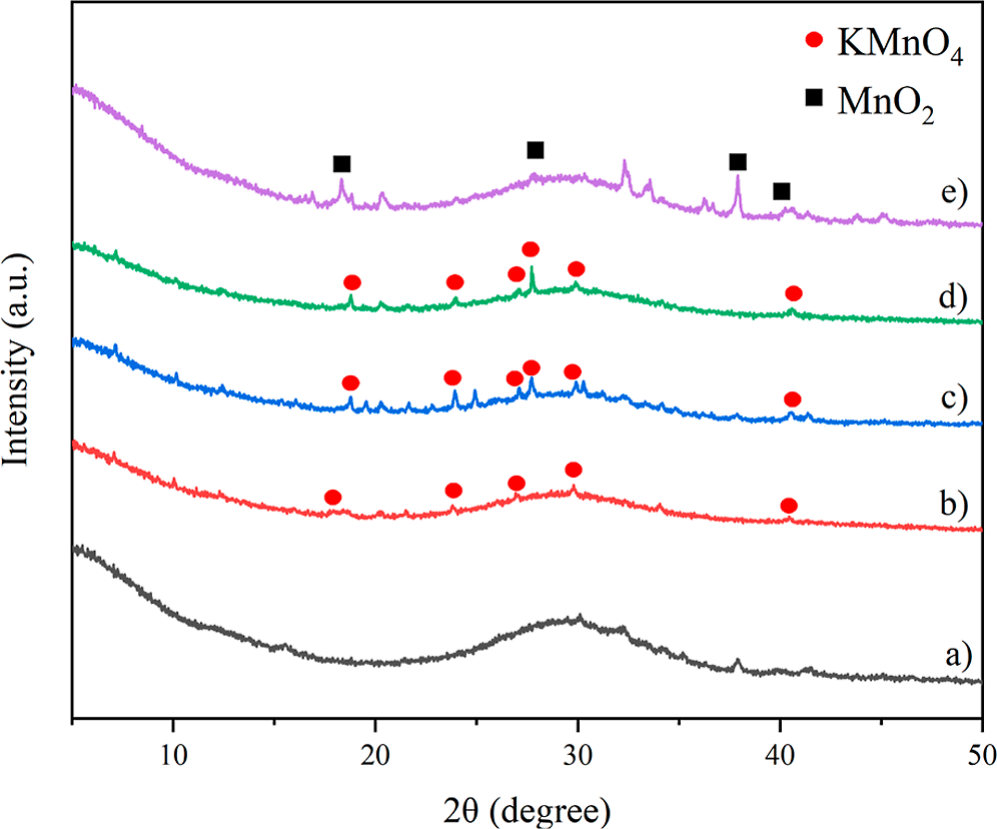
XRD patterns of (a) fresh SC-ASA, (b) fresh 2KM/SC-ASA, (c) fresh 4KM/SC-ASA, (d) fresh 6KM/SC-ASA, and (e) spent 4KM/SC-ASA.

The results of the X-ray diffraction (XRD) analysis of the SC-ASA and the fresh and spent *X*KM/SC-ASA samples, where X represents the KMnO_4_ loading level (% w/w) on SC-ASA and KM denotes KMnO_4_, are displayed in Figure 1. The diffraction patterns of all samples showed a broad peak for 2θ in the range 20–40°, which was characteristic of amorphous SiO_2_.^30^ The XRD patterns of the samples containing KMnO_4_ appeared at 2θ = 18.76, 23.93, 26.00, 27.08, 27.74, 29.89, and 40.52° (JCPDS: 89-3951).^31^ However, 2KM/SC-ASA showed a poorly crystalline KMnO_4_ structure (Figure 1b). The XRD peaks of the crystalline KMnO_4_ phase increased with increased KMnO_4_ loading. An obvious crystalline KMnO_4_ phase in the XRD patterns has been reported for amorphous SiO_2_.^32^ Using the impregnation method, these results confirmed the successful loading of KMnO_4_ on the SC-ASA.

After C_2_H_4_ adsorption, the XRD peaks of KMnO_4_ disappeared, as shown in Figure 1e. Additionally, for 4KM/SC-ASA, new XRD peaks were observed at 2θ = 18.31, 27.84, 37.86, and 40.29°, corresponding to MnO_2_ (JCPDS: 44-0141).^33,34^ The MnO_2_ occurred from reducing KMnO_4_ with the ethylene compound, which also released potassium hydroxide (KOH), CO_2_, and H_2_O, as shown in eq 1(12)
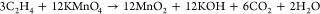
1

#### 2.2.2 Textural Properties Analysis

**Figure 2:**
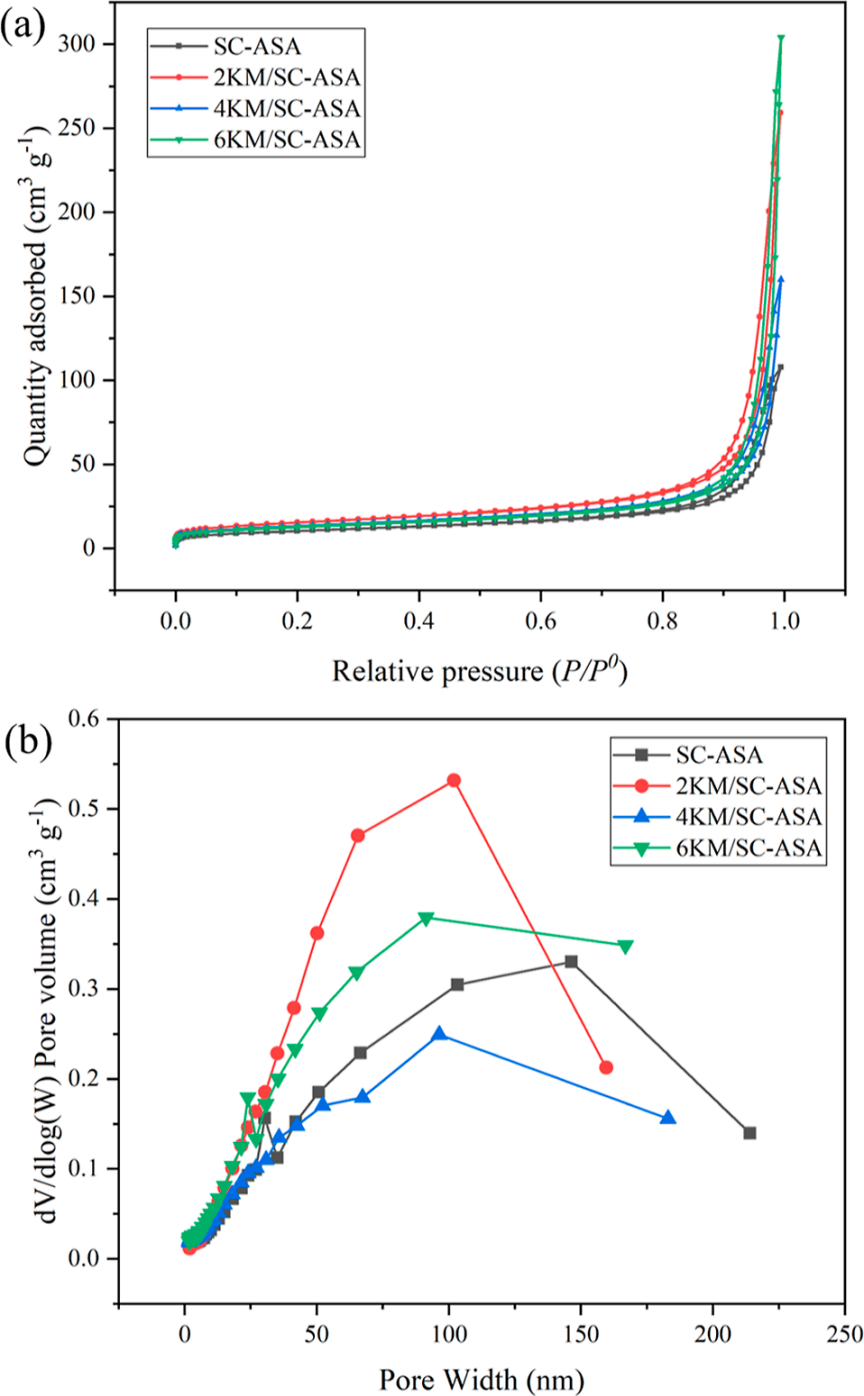
(a) N_2_ adsorption–desorption isotherms and (b) pore size distribution of all samples.

The specific surface area and pore texture of all samples were examined by using a N_2_-sorption analyzer, as shown in Figure 2 and Table 2. According to the International Union of Pure and Applied Chemistry classified isotherms, the N_2_ adsorption–desorption isotherms of every sample in Figure 2a showed a quantity of weakly absorbed capacity of a Type IV isotherm with a small hysteresis loop at high relative pressure (*P*/*P*_0_ > 0.80), suggesting that the mechanism of pore filling and emptying was through capillary condensation in the mesoporous structure.^35^ In Figure 2b, the pore size distribution curves of SC-ASA and impregnated SC-ASA exhibit a wide range between 0 and 250 nm, with a dominant pore size of approximately 100 nm. Furthermore, increasing the KMnO_4_ loadings resulted in decreases in the specific surface area, average pore size, and total pore volume, as shown in Table 2. The specific surface area of the samples decreased from 58.20 to 42.70–46.10 m^2^ g^–1^ when the different amounts of KMnO_4_ (2–6% w/w) were loaded on the SC-ASA, potentially because of the deposition of KMnO_4_ on the surface of the SC-ASA.^36^ The average pore size and total pore volume of SC-ASA decreased with an increase in the KMnO_4_ concentration from 2 to 6% w/w, as shown in Table 2. The reduction in the average pore size and total pore volume might have been due to the KMnO_4_ impregnated on SC-ASA, which could have resulted in the formation of free KMnO_4_ that covered or aggregated on the SC-ASA surface, leading to pore-blocking and hindering the efficacy of the internal surface for N_2_ gas adsorption.

**Table 2:** Textural Properties of SC-ASA before and after Impregnation with KMnO_4_

sample	surface area (m^2^ g^–1^)	pore volume (cm^3^ g^–1^)	pore diameter(nm)
SC-ASA	58.20	0.36	24.80
2KM/SC-ASA	53.60	0.30	25.80
4KM/SC-ASA	45.80	0.28	23.00
6KM/SC-ASA	43.40	0.25	21.60

#### 2.2.3 Scanning Electron Microscopy Image and Energy Dispersive X-ray Analyses

**Figure 3:**
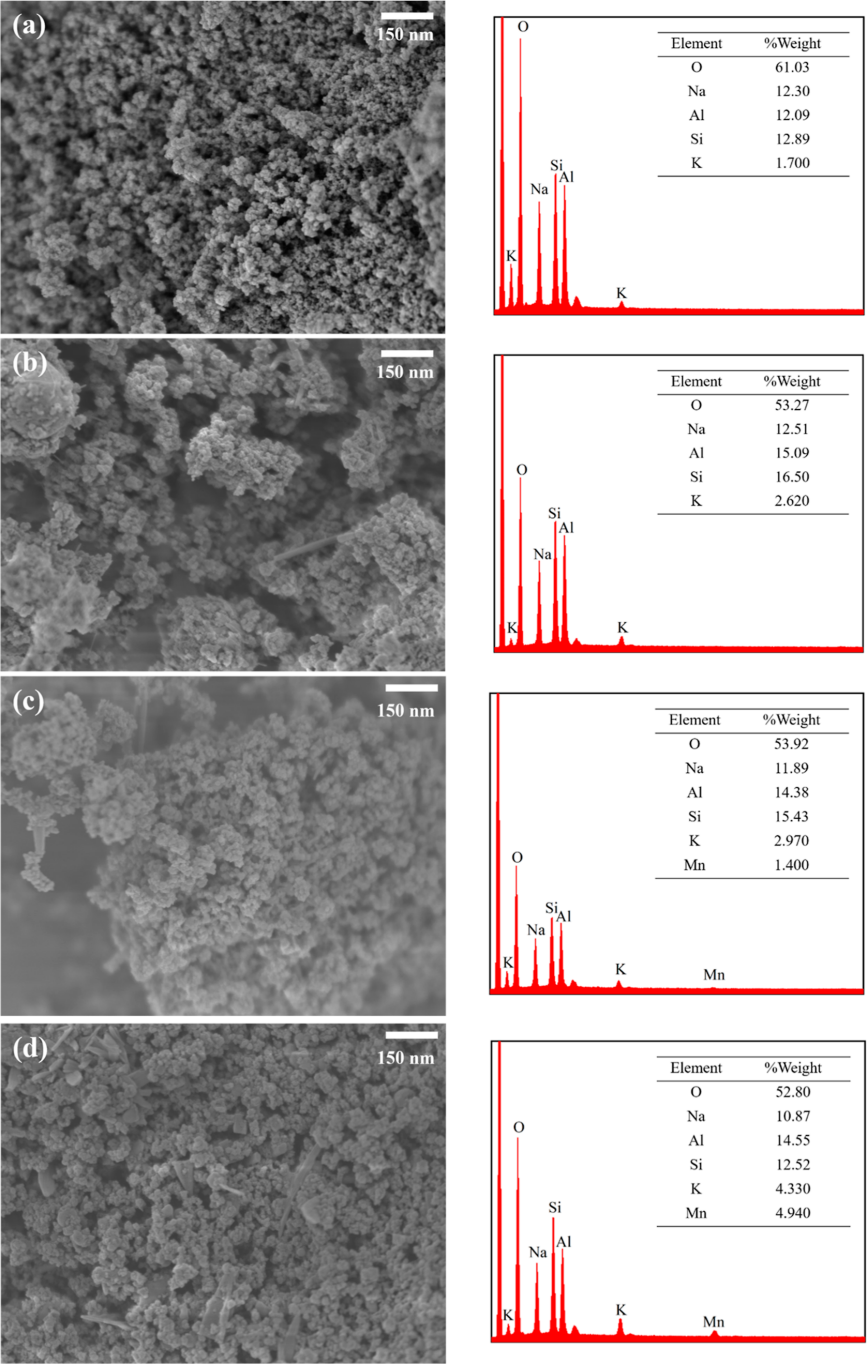
SEM images with EDS results for (a) SC-ASA, (b) 2KM/SC-ASA, (c) 4 KM/SC-ASA, and (d) 6 KM/SC-ASA.

The morphological changes of SC-ASA and *X*KM/SC-ASA were imaged by using scanning electron microscopy (SEM), as shown in Figure 3. The SEM image of SC-ASA (Figure 3a) shows agglomerated and spherical particles with an average particle size of 43.94 ± 9.75 nm. After impregnation with KMnO_4_, larger agglomerated particles were observed, with average particle sizes of 49.10 ± 8.80, 57.56 ± 12.72, and 59.74 ± 9.99 nm for 2KM/SC-ASA (Figure 3b), 4KM/SC-ASA (Figure 3c), and 6KM/SC-ASA (Figure 3d), respectively. Some particles having a needle-rod shape, characteristic of KMnO_4_ crystal particles, were observed in the samples with KMnO_4_ loading.^37^

The quantitative and qualitative numbers of the main elements in the samples were analyzed based on energy dispersive X-ray analysis (EDS), as shown in Figure 3, and Supporting Information: the EDS-mapping images (Figure S1). The primary components of SC-ASA (O, Na, Al, Si, and K) were present in all samples. However, manganese (Mn) was not visible in the 2KM/SC-ASA sample, presumably because the majority of the KMnO_4_ was deeply embedded inside the support’s pores; nonetheless, it was observed at 1.40 and 4.94% w/w in the 4KM/SC-ASA and 6KM/SC-ASA samples, respectively. Nevertheless, the amounts of K increased with an increase in the KMnO_4_ loadings, confirming the desired higher loading of KMnO_4_ on the support.

### 2.3 Effect of KMnO_4_ Loading on C_2_H_4_ Scavenging

The C_2_H_4_ scavenging ability of *X*KM/SC-ASA was measured for 450 min, based on the remaining C_2_H_4_ concentration in the headspace of the test flask. The KMnO_4_ uptakes of the different *X*KM/SC-ASA samples are presented in Table 3. The uptake of KMnO_4_ in the samples increased to 86.9, 444.8, and 526.4 mg 100 g^–1^ at 2, 4, and 6% w/w KMnO_4_ loading, respectively.

**Table 3:** KMnO_4_ Uptake and C_2_H_4_ Removal Capacity and Efficiency of SC-ASA with Different KMnO_4_ Loadings at 120 min of Contact Time

		C_2_H_4_ removal at 120 min contact time	
sample	KMnO_4_ uptake (mg 100 g^–1^)	capacity (mg g^–1^)	efficiency (%)
2KM/SC-ASA	86.9	92.7	18.8
4KM/SC-ASA	444.8	472.0	100.0
6KM/SC-ASA	526.4	434.0	89.5

**Figure 4:**
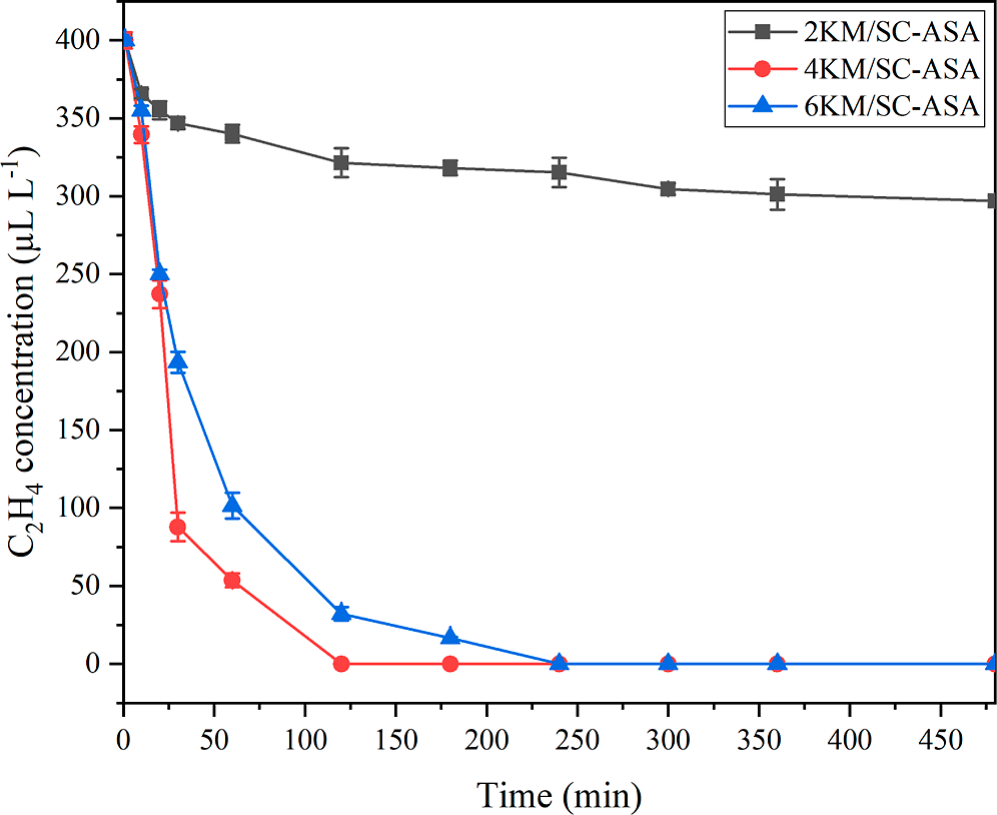
Effect of KMnO_4_ loading on C_2_H_4_ adsorption. Conditions: 0.5 g of sorbent, 400 μL L^–1^ of ethylene, 25–27 °C, and 70–75% RH.

The relationship between the remaining C_2_H_4_ concentration and the time is shown in Figure 4. Considering the C_2_H_4_ adsorption capacity and efficiency of KM/SC-ASA at 0, 2, 4, and 6% w/w, both KMnO_4_ uptakes and morphology changes on SC-ASA have a large effect on the C_2_H_4_ adsorption performance, as shown in Table 3.^21^ The C_2_H_4_ concentrations in the flasks containing 4KM/SC-ASA and 6KM/SC-ASA decreased from the initial concentration (400 μL L^–1^) to below 0.05 μL L^–1^. In contrast, with 2KM/SC-ASA, the remaining C_2_H_4_ concentration was still high at 297.0 μL L^–1^ after 360 min. Furthermore, in the 120 min experiment, the 4KM/SC-ASA sample also had the highest C_2_H_4_ adsorption capacity and efficiency among the other adsorbents. At higher KMnO_4_ uptakes (6KM/SC-ASA), the C_2_H_4_ adsorption capacity and efficiency values were reduced because the excessive KMnO_4_ loading could cover and agglomerate on the SC-ASA surface, leading to blockage of the pores and hindering the efficacy of C_2_H_4_ diffusion. Thus, its surface area and the reactive group on SC-ASA might have favored C_2_H_4_ adsorption and accounted for the decreased C_2_H_4_ concentration with increased KMnO_4_ loading. Several reports on the irreversible adsorption-oxidation mechanism of KMnO_4_-based porous materials are associated with a porous material that physiologically adsorbed C_2_H_4_. KMnO_4_ oxidizes its double bond and is broken into CO_2_ and H_2_O.^18,22^ Additionally, KMnO_4_ is a wide-spectrum oxidizing agent that reacts with C_2_H_4_ gas, an irreversible color change from purple (MnO_4_^–^) to brown (MnO_2_).^18^ This phenomenon is noted in Supporting Information: an irreversible color change of KMnO_4_ (Figure S2), which shows that an irreversible color change characteristic of KMnO_4_ is a C_2_H_4_ gas indicator for determining fruit ripeness. Shin et al. (2023) developed a sensitive C_2_H_4_ gas indicator using KMnO_4_-based packaging film by checking its color change as a function of the amount of C_2_H_4_ gas and time of exposure for optimal ripeness determination in kiwifruit packaging.^38^ Therefore, 4KM/SC-ASA had the best C_2_H_4_ scavenging performance and was the most effective C_2_H_4_ scavenger in this study; consequently, it can be used not only to prolong the shelf life of fruits but also as a C_2_H_4_ indicator to predict fruit ripeness.

### 2.4 Kinetic Study

**Figure 5:**
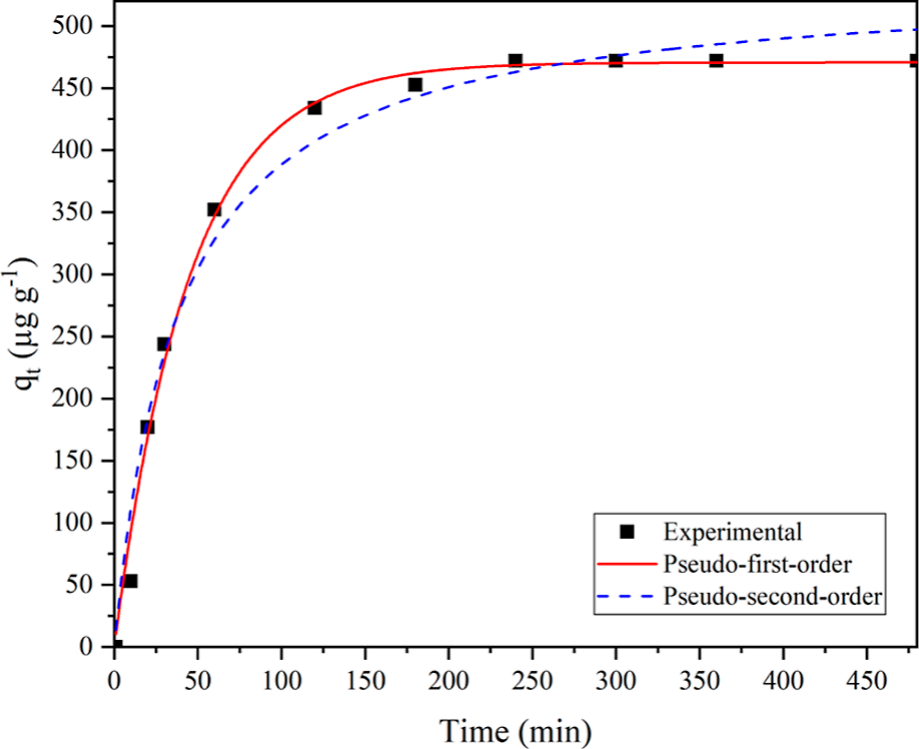
Fitted curves of pseudo-first-order and pseudo-second-order kinetic models.

Adsorption kinetics are essential for evaluating adsorption efficiency and identifying the adsorption mechanism, chemical reaction rate, and potential rate-controlling steps. The experimental data of the C_2_H_4_ removal using 4KM/SC-ASA were fitted to two kinetics models (pseudo-first-order and pseudo-second-order), as shown in Figure 5. The most suitable kinetic model was evaluated based on the *R*^2^ value, which is used to indicate how well a statistical model fits the data provided. The parameters *k* and *q*_e_ indicate the removal rate and the amount of C_2_H_4_ removal at equilibrium for the samples, respectively. The kinetic parameters (*q*_e_ and *k*) and the statistical test parameters [*R*^2^ and sum squares of error (SSE)] after the model fitting for every sample are presented in Table 4. The *R*^2^ value of the pseudo-first-order kinetic model (0.9932) was greater than that for the pseudo-second-order kinetic model (0.9814), indicating that the pseudo-first-order kinetic model provided a better fit of the adsorption behavior than the pseudo-second-order kinetic model. This suggested that the dominant adsorption behavior in C_2_H_4_ adsorption is physisorption. However, both the *R*^2^ values were greater than 0.9000, indicating that physical and chemical adsorptions coexist in the system.^36^ In addition, the SSE values were slightly lower for the pseudo-first-order model than for the pseudo-second-order kinetic model. These results could imply that the C_2_H_4_ adsorption occurred in multiple steps, involving gas diffusion through the interface, surface adsorption, and chemical reaction between the adsorbent and adsorbate.

**Table 4:** Parameters of Kinetic Models for C_2_H_4_ Adsorption onto 4KM/SC-ASA

pseudo-first order				pseudo-second order			
*q*_e_ (μg g^–1^)	*k* (min^–1^)	*R*^2^	SSE	*q*_e_ (μg g^–1^)	k (min–1)	*R*^2^	SSE
470.7	0.020	0.9932	1.197	536.7	4.880	0.9814	1.292

### 2.5 Effects of C_2_H_4_ Scavenger on Mango Quality

**Figure 6:**
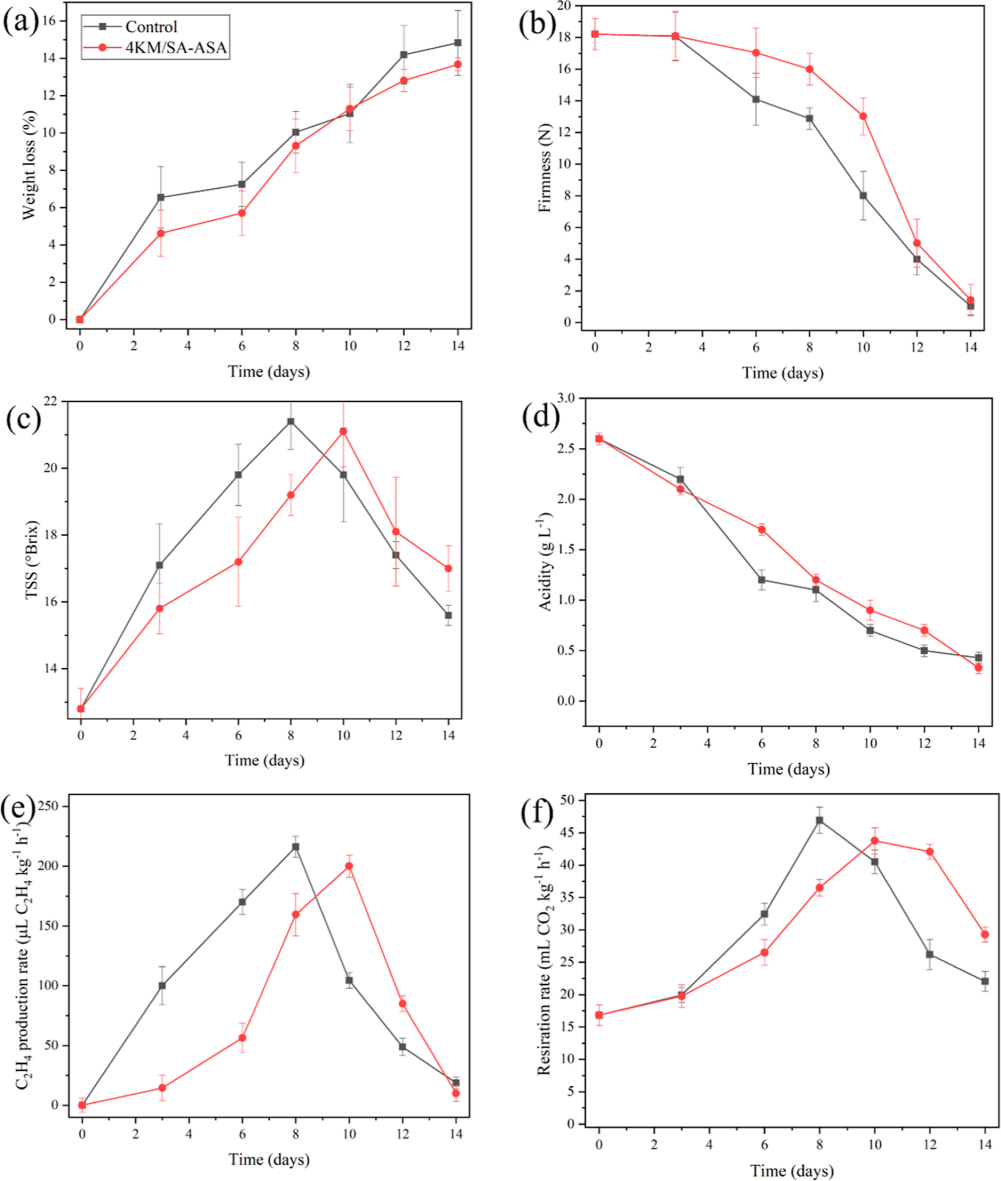
Effects of C_2_H_4_ scavenger on (a) weight loss, (b) firmness, (c) TSS, (d) acidity, (e) C_2_H_4_ production rate, and (f) respiration rate during storage under ambient conditions.

4KM/SC-ASA had the highest C_2_H_4_ removal efficiency. Therefore, it was chosen to study its effect on mango quality during storage. The data regarding the ripening days of mango are presented in Figure 6. Four sachets containing 5 g of 4KM/SC-ASA per sachet were packed in each cardboard corrugated box containing 1.6 kg of mango fruits and stored under ambient conditions (24–25 °C and 70–80% RH) to maintain the shelf life of mango fruits up to 14 days.

#### 2.5.1 Physiological Weight Loss

Weight loss is one of the most critical determinants of mango storage life. Ethylene action increases the respiration rate and causes fruit water loss through transpiration or evaporation, leading to configurational changes in the appearance and textural and nutritional qualities during the ripening process.^18^Figure 6a shows the relationship between the weight loss of mango fruits and the storage time when using the ethylene scavenger (4KM/SC-ASA) in the packaging. The results indicated that the weight loss of the mango fruits in the boxes with and without 4KM/SC-ASA increased during the storage period. Nevertheless, the weight loss of the mango fruits packed with 4KM/SC-ASA (13.68%) was significantly lower than that for those without the ethylene scavenger (14.83%) after 14 days of storage. The weight loss during fruit storage was mainly due to water loss and physiological processes such as transpiration, respiration, and configurational changes of plant tissues during senescence.^22,39^ The lower weight loss percentage with the 4KM/SC-ASA was because KMnO_4_ is an oxidative agent that degrades C_2_H_4_ to H_2_O and CO_2_. The accumulation of H_2_O and CO_2_ led to increased humidity in the boxes, resulting in slower moisture loss and respiration rate of the mango fruits.^23^

#### 2.5.2 Firmness

Nam Dok Mai mangoes are commonly harvested during their mature stage and then become soft as ripening proceeds during storage. For these mango fruits, texture is one of the most important quality attributes, influenced by firmness and internal flesh change. The current results revealed that the firmness of the mangoes decreased gradually for mango fruits packed with or without the C_2_H_4_ scavenger during the storage period, as shown in Figure 6b. Since the presence of C_2_H_4_ could promote the activities of cell-wall-degrading enzymes, including galactosidase, pectin methylesterase, and polygalacturonase, the cross-linking polysaccharides in the cell wall disintegrate.^6,40^ Moreover, an increase in fruit firmness could have resulted from insoluble pectin turning into water-soluble pectin due to the protopectin maturation process.^41^ In addition, it is associated with respiration because the carbohydrate metabolism converts sugar and oxygen (O_2_) into energy, H_2_O, and CO_2._^40^ However, the C_2_H_4_ scavenger positively affected mango firmness over storage time. During the first 6 days of storage, the firmness of the mango fruits packed with the C_2_H_4_ scavenger decreased from 18.21 to 17.04 N, while the firmness of the control mango fruits sharply decreased to 14.10 N. Moreover, the loss in the firmness of mango fruits packed with the C_2_H_4_ scavenger was approximately 22.57% lower than those in the control treatment (6.425%). The C_2_H_4_ scavenger reduced C_2_H_4_ production and the respiration rate.^40,42,43^ These results were similar to those of Fatima et al. (2023) who reported that KMnO_4_ could adsorb ethylene and slow the respiration rate and ripening process, reducing the decline in mango firmness during the storage period.^6^

#### 2.5.3 Total Soluble Solid and Acidity Changes

The acidity and TSS contents are essential quality parameters used to evaluate the taste of the fruit produced, as shown in Figure 6c,d, respectively. Typically, TSS increases and acidity decreases during the storage period due to the conversion of starch to sugars and the utilization of organic acids by metabolic processes.^23^

The mango TSS value on the first observation day was 14.50°Brix. The TSS values of the mango fruit samples packed with and without the C_2_H_4_ scavenger were 21.10 and 19.80°Brix, respectively, in the first 10 days and then slightly decreased to 17.00 and 15.60°Brix, respectively, on day 14. Other researchers also found that the TSS of mangoes increased to the maximum at the fully ripe stage and then slightly decreased toward the senescence stage.^40,44^ As shown in Figure 6c, the mango fruits packed with 4KM/SC-ASA had lower TSS values than the control treatment, suggesting that KMnO_4_ could delay the ripening and senescence because of its ability to remove ethylene, leading to reductions in several enzymatic activities in the hydrolysis of insoluble polysaccharides into simple sugars.^40^ The water loss also caused a rise in the amount of sugar in the fruit during storage.

At the commencement of storage, the acidity value of the mango fruits was 2.600 g L^–1^. Both acidity values (with and without the C_2_H_4_ scavenger) gradually decreased during storage. The reduction in the acidity values might have been due to the organic acids (such as citric acid and malic acid) in the fruits directly used in the citric acid cycle.^40^ Furthermore, the highest acidity values were reported during the unripe stage, with a decrease during the ripening of mango fruits.^45−47^ In Figure 6d, the acidity values decreased faster in the mango fruits packed with the C_2_H_4_ scavenger than in the control treatment. Therefore, using KMnO_4_ resulted in a slow decay of mango acidity during storage, extending the ripening process. The C_2_H_4_ oxidation by KMnO_4_ released CO_2_ as a byproduct, slowing down the fruit’s respiration rate, with the accumulated concentration of CO_2_ leading to carbonic acid formation, which slows fruit acidity decay.^6^

#### 2.5.4 C_2_H_4_ Production and Respiratory Rate

Since C_2_H_4_ released from fruits can promote respiration, the fruit quality characteristics change in their chemical composition, appearance, and texture, leading to fruit ripening and senescence. Table 5 presents the C_2_H_4_ and CO_2_ production rates in the headspace of the mango storage containers during storage (0–14 days) with and without the C_2_H_4_ scavenger at 24–25 °C and 70–80% RH.

**Table 5:** C_2_H_4_ and CO_2_ Production Rates by Mangoes Stored at 24–25 °C and 70–80% RH

	C_2_H_4_ production rate (μL kg^–1^ h^–1^)		CO_2_ production rate (mL kg^–1^ h^–1^)	
storage time (days)	control	4KM/SC-ASA	control	4KM/SC-ASA
0	0.047	0.047	16.83	16.83
3	100.1	14.51	19.93	19.76
6	170.1	56.44	32.43	26.51
8	216.2	159.6	46.94	36.52
10	104.5	200.1	40.52	43.76
12	48.92	85.12	26.19	42.08
14	18.76	10.33	22.03	29.29

The C_2_H_4_ production in both treatments continued to increase from day 1 to days 8–10 of storage and then gradually decreased until the end of storage, as shown in Figure 6e. The maximum C_2_H_4_ production rates of the mango fruits in the control and C_2_H_4_ scavenger treatments were 216.2 μL of C_2_H_4_ kg^–1^ h^–1^ on day 8 and 200.1 μL of C_2_H_4_ kg^–1^ h^–1^ on day 10. Therefore, the mango fruits packed in the box with the C_2_H_4_ scavenger eliminated more C_2_H_4_ than those in the control treatment during storage. After the maximum C_2_H_4_ production, both rates considerably decreased in the ripe stage. The different maturity stages of the mango fruits affected the ethylene removal performance of the adsorbent. Bhutia et al. (2011) suggested that the KMnO_4_-based adsorbent could delay the C_2_H_4_ and CO_2_ production rates in the mature stage and half-ripe mango fruits.^48^ Therefore, using the C_2_H_4_ scavenger could reduce the rate of C_2_H_4_ production and respiration in the half-ripe and mature stages of mango fruits.

The CO_2_ production rate was used to determine the respiration rate in both treatments. On the first day of observation, the mango fruits packed with and without the C_2_H_4_ scavenger had the same CO_2_ production rate (16.83 mL of CO_2_ kg^–1^ h^–1^). As shown in Figure 6f, the CO_2_ production rates of the mango fruits packed with and without the C_2_H_4_ scavenger increased during the first 10 (43.76 mL of CO_2_ kg^–1^ h^–1^) and 8 (46.94 mL of CO_2_ kg^–1^ h^–1^) days of the storage period, respectively, followed by a decrease as the storage time increased further. Moreover, the CO_2_ production rate of the mango fruits packed with the C_2_H_4_ scavenger was lower than that in the control treatment during the first 10 days. Thus, KMnO_4_ delayed the respiration rate of mango fruits, thereby extending their shelf life. These results were consistent with the research by Shenoy et al. (2022), who found that using KMnO_4_ could delay the respiration rate due to increased CO_2_ and decreased O_2_ concentrations initially before reaching equilibrium.^39^ In addition, Elzubeir et al. (2018) reported that the accumulated CO_2_ rises in a fruit box from an oxidation reaction between KMnO_4_ and C_2_H_4_, inhibiting the fruit respiration rate.^49^

**Figure 7:**
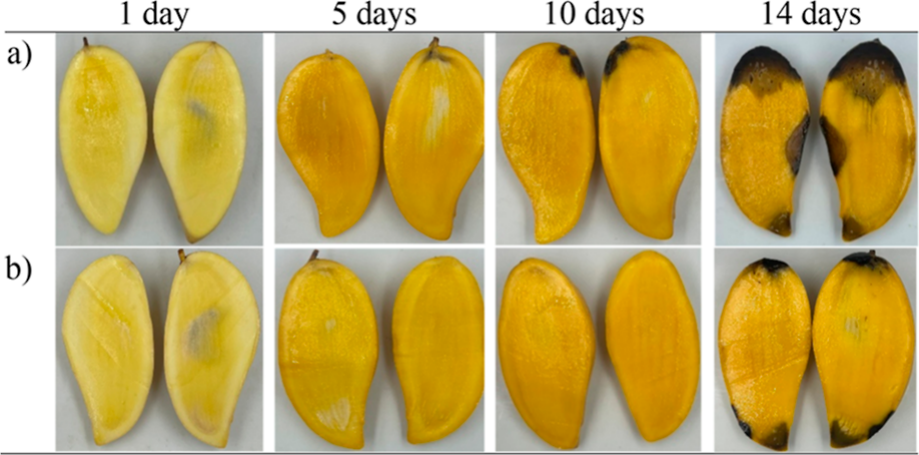
Photographs of internal flesh changes of mango fruits packed (a) without and (b) with sachets of 4KM/SC-ASA stored under ambient conditions (24–25 °C and 70–80% RH) after 1, 5, 10, and 14 days.

The loss of flesh color and flesh browning were investigated in the mango fruits packed with and without the C_2_H_4_ scavenger on days 1, 5, 10, and 14 of storage, as shown in Figure 7. On day 1, the mango fruits in both treatments were not ripe, and the flavor would probably disappoint consumers due to the high levels of starch and acids, resulting in a low soluble sugar content.^50^ The internal flesh color of the mango fruit changed from pale yellow to deep golden yellow during storage, which might have been due to the disappearance of chlorophyll and the appearance of other pigments, such as anthocyanins and carotenoids.^51^ On day 5 of storage, the mango fruits packed with the C_2_H_4_ scavenger had a desirable, healthy pulp (soft, ripe, and flavorful taste and aroma), while the mango fruits in the control treatment presented a jelly seed pulp, with the healthy flesh of mango fruit that is desirable for consumer acceptance.^7^ Additionally, when mango fruits ripen, their physiological, biochemical, and structural characteristics change. These changes include water loss, hydrolysis of starch into sugars, and increased activity of several enzymes, such as α-amylase.^7,50^ Rama Krishna et al. (2020) revealed that the jelly seed fruit had a higher respiration rate and reduced levels of β-carotene and total antioxidant capacity, leading to a loss of nutritional value, while the C_2_H_4_ production rate was unchanged.^4^ In addition, there was a delay in the appearance of darkening and rotting spots on the mango fruits with the C_2_H_4_ scavenger compared to that for the mango fruits in the control treatment on days 10 and 14, respectively. Maldonado-Celis et al. (2019) reported that the infection of mango was generated by anthracnose in mango tissues caused by fungal species of *Colletotrichum* (commonly *C. siamese* and *C. asianum*) that affected the appearance and resulted in losses in terms of quality and quantity.^7^

Typically, at ambient temperature, mango fruits ripen very quickly after detaching from the tree in approximately 3–6 days, becoming overripe and spoiled within 10 days.^44,46^ Therefore, the current study showed that packing mango with the C_2_H_4_ scavenger delayed chemical and physiological changes, resulting in a prolonged shelf life.

## 3 Conclusions

The developed C_2_H_4_ scavenger, consisting of SC-ASA and loaded with KMnO_4_, can be considered not only as a favorable C_2_H_4_ gas scavenger but also as suitable for maintaining fruit quality characteristics during storage. The results revealed that 4% w/w KMnO_4_ on ASA derived from SC (4KM/SC-ASA) produced the greatest ethylene reduction. In addition, the ethylene removal kinetics of 4KM/SC-ASA evaluated in this study conformed to a pseudo-first-order kinetic model. Furthermore, 4KM/SC-ASA was utilized as a C_2_H_4_ scavenger in storage studies on postharvest mango fruits. It effectively reduced C_2_H_4_ production, respiration rates, and quality changes of the mangoes during their extended storage. These findings should contribute not only to the development of a C_2_H_4_ scavenger to reduce postharvest losses but also to the reusability of waste from the sugar and ethanol industries as a value-added material.

## 4 Materials and Methods

### 4.1 Preparation of Amorphous SC-ASA

For the preparation of SC-ASA, 10 g of SC was mixed with 2 M sodium hydroxide (NaOH) and made up to 60 mL before heating at 70–80 °C for 24 h to obtain a solution of sodium silicate (Na_2_SiO_3_). The Na_2_SiO_3_ solution was separated using centrifugation at 8000 rpm for 10 min and passed through a filter paper (Whatman no. 41) and denoted as Solvent I. After that, 5.265 g of sodium aluminate (NaAlO_2_) was dissolved using 32 mL of deionized (DI) water and stirred until the solution was homogeneous and denoted as Solvent II. Then, Solvent I was dropped into Solvent II to obtain gels. Next, the mixture was transferred to a Teflon-lined autoclave, which was hydrothermally heated to 80 °C for 6 h. Finally, the obtained white sediment was filtered, washed with DI water until the pH was below 9, and dried at 80 °C overnight.

### 4.2 Loading of KMnO_4_ on SC-ASA

The ethylene scavenger (1.0 g) was generated by immersing SC-ASA in a solution of KMnO_4_ at 2, 4, or 6% w/w for 24 h at room temperature. The impregnated SC-ASA was dried in a vacuum oven at 60 °C for 48 h. The impregnated samples were denoted as *X*KM/SC-ASA, where *X* represents the KMnO_4_ loading level (% w/w) on SC-ASA and KM denotes KMnO_4_. The obtained *X*KM/SC-ASA samples were stored in a dry, dark, tightly closed container.

### 4.3 C_2_H_4_ Adsorption Experiments

The ability of *X*KM/SC-ASA to remove C_2_H_4_ was evaluated using a closed system that contained a C_2_H_4_ concentration of 400 μL L^–1^ (400 ppm). Each sample (0.5 g) was placed in a sealed 500 mL flask under ambient conditions (24–25 °C and 70–80% relative humidity). Then, 200 μL of C_2_H_4_ gas (99.999%, Linde, Thailand) was injected into the sealed flask through a septum. To determine the C_2_H_4_ concentration, a headspace gas sample (1 mL) was withdrawn from the sealed flask through the septum using a 1 mL gastight syringe every 30 min for 8 h. The withdrawn gas sample was investigated using gas chromatography (GC; Shimadzu GC-14A; Japan) equipped with a flame ionization detector (FID) and a Porapak Q, 80/100 mesh column. The calibration data were successfully fitted to a linear trendline with a coefficient of determination (*R*^2^) of 0.9992.

C_2_H_4_ adsorption capacity was quantified by calculating the ethylene adsorption amount as defined by the following^35^
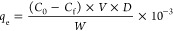
2where *q*_e_ is the amount of C_2_H_4_ gas adsorbed on the sample (mg g^–1^), *C*_0_ and *C*_f_ are the initial and final C_2_H_4_ concentration (μL L^–1^), respectively, *V* is the volume of flask (L), *D* is the density of C_2_H_4_ (1.18 g mL^–1^), and *W* is the weight of sample (g).

### 4.4 Characterization

The *X*KM/SC-ASA samples, where *X* is 2, 4, or 6, were characterized by using the following techniques to obtain their physical and chemical properties.

#### 4.4.1 X-ray Powder Diffraction

The crystal properties of the samples were characterized using X-ray powder diffraction (XRD; Bruker D8 Advance) equipped with CuKα (λ = 1.5406 Å) radiation. The intensity data were recorded for an angular 2θ range of 5–60° with a scanning rate of 0.04° per second. The working voltage and the instrument’s current were 40 kV and 40 mA, respectively.

#### 4.4.2 N_2_ Adsorption–Desorption

The average specific surface area, pore diameter, and pore size of the samples were determined using a N_2_-sorption analyzer (Autosorp-1C; Quantachrome Instruments; USA). The specific surface area and the pore size and volume were all determined by using the Barrett–Joyner–Halenda method. The N_2_ adsorption–desorption data were gathered in liquid N_2_ at −196 °C. Prior to each measurement, degassing was performed at 300 °C for 3 h using an Autosorp degasser (model AD-9).

#### 4.4.3 XRF Spectroscopy

SiO_2_ and other components in the SC and SC-ASA were determined by using XRF (Horiba, Mesa 500; Japan) with a 15/50 keV X-ray tube.

#### 4.4.4 UV–vis Spectroscopy

The adsorption properties of the samples at a wavelength of 527 nm were analyzed using a UV–vis spectrophotometer (Cary WinUV; Varian; Australia). For a typical sample preparation, the adsorbent (0.2 g) was placed in a 100 mL volumetric flask, filled with DI water to 100 mL, and covered with foil. Then, it was shaken for 2 min and centrifuged (J2-MC; Beckman; USA) for 45 min at 2000 rpm.

#### 4.4.5 Scanning Electron Microscopy with Dispersive X-ray Spectroscopy

The morphological properties and surface structure of the samples were examined using SEM with EDS (SEM/EDS; S-500; Hitachi Ltd.; Japan). Prior to the SEM/EDS measurement, the powder sample was sprinkled on a carbon sticky tab of an aluminum specimen mount and coated with a thin (<10 nm) gold (Au) layer using a sputter coater (Edwards Laboratories, USA) at 50–60 mTorr pressure for 50 s.

### 4.5 Kinetics of Ethylene Removal

Two kinetic models (pseudo-first-order and pseudo-second-order) were used to fit the ethylene adsorption data of 4KM/SC-ASA to determine the best kinetic model to describe the ethylene removal behavior and to evaluate the ethylene mass transfer efficiency to the adsorbent (*q*_e_) and the potential rate-controlling step (*k*). The nonlinearized forms of the pseudo-first-order and pseudo-second-order kinetic models are generally represented in eqs 3 and 4, respectively^35^
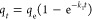
3
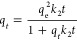
4where *q*_e_ and *q*_*t*_ are the amounts of ethylene removal per mass of the adsorbent at equilibrium (μg g^–1^) and time, respectively, and *k*_1_ and *k*_2_ are the rate constants of the pseudo-first-order (min^–1^) and pseudo-second-order kinetic model (μg g^–1^ min^–1^), respectively.

The fitness of the adsorption data was further analyzed by calculating the SSE using eq 5(52)
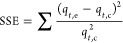
5where *q*_*t*,e_ and *q*_*t*,c_ are the experimental and calculated adsorption capacities (μg g^–1^), respectively.

The best set of parameters had minimal SSE between the experimental and calculated values.

### 4.6 Storage of Fruit Using Ethylene Scavenger-Based Sachets

Golden Nam Dok Mai mango fruits were harvested at their mature stages (80–90 days old) from the local orchards in Chaiyapoom province, Thailand, in June 2023. Mango samples without defects were selected and also for their weight, shape, and peel color uniformity. After checking for disease and mechanical damage, the selected mango samples were desapped, prewashed with sodium hypochlorite solution, and dried under the fan until dry. In each corrugated fiberboard five-ply box (20 × 30 × 20 cm), four mango fruits (weighing approximately 1.6 kg in total) were placed in separate samples, with and without four sachets (5 g of ethylene scavenger per sachet). All fruit samples were stored under ambient conditions (24–25 °C and 70–80% RH). The physiological and physicochemical properties of the sample fruits were evaluated during 0–14 days.

#### 4.6.1 C_2_H_4_ and CO_2_ Production

The C_2_H_4_ and CO_2_ productions of the fruit samples were analyzed every 3 days during storage. Eight mango fruits (each weighing approximately 380–460 g) were chosen, and each one was placed in a gastight polypropylene container with a lid (16 × 21.7 × 9.2 cm). Gas samples (1 mL) were collected using a gastight syringe from the headspace atmosphere and determined using the GC equipped with the FID and a Porapak Q, 80/100 mesh column (to evaluate the C_2_H_4_ production rate), a thermal conductivity detector, and a Unibead C, 80/100 mesh column (to determine the respiration rate or the CO_2_ production rate). The C_2_H_4_ and CO_2_ production rates were expressed as μL C_2_H_4_ kg^–1^ h^–1^ and mL CO_2_ kg^–1^ h^–1^, respectively.^4^

#### 4.6.2 Weight Loss Evaluation

The weight loss of the fruit samples with and without the synthesized adsorbent in the packaging was measured on the initial day and each subsequent sampling day during storage.^6^ The percentage weight loss of the mango fruits was calculated using eq 6
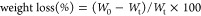
6where *W*_0_ is the initial weight before storage and *W*_t_ is the weight after storage.

#### 4.6.3 Total Soluble Solids and Acidity Evaluation

The TSS and the acidity values were measured from the mango juice using a digital refractometer (PAL 1; Atago Co. Ltd.; Japan) and expressed in degrees Brix (°Brix) and in terms of the predominant acid equivalents (citric acid and malic acid, in g L^–1^), respectively. For typical sample preparation, 30 g of pulp tissue was ground with 90 mL of DI water using a mortar, followed by filtration by passing through a filter paper (Whatman no. 40) to obtain the mango juice.^44^

#### 4.6.4 Firmness

The firmness of the mango fruits was determined using a texture analyzer (TA.XTplusC; Stable Micro Systems; UK) equipped with a cylindrical stainless probe (6 mm in diameter), a 50 kg load cell, and a test speed of 0.2 mm s^–1^. The fruit samples were placed in the center of the platform and measured three times at three longitudinal points on each sample. The data were recorded as the average reading. The firmness results were expressed in terms of the maximum force (N).^47^

### 4.7 Statistical Data Analysis

Each experiment was conducted in triplicate. The experimental data were subjected to analysis of variance (ANOVA) using Statistica 8.0 software (StatSoft; USA). The statistical significance of the differences between mean values was assessed at *p* ≤ 0.05, and Duncan’s new multiple range test was used for all statistical analyses.

## Supporting Information Available

EDS-mapping of elements on the surface of samples and images of color changes in the C_2_H_4_ reduction (PDF)
