# Supplementary material for: Potassium Permanganate-Impregnated Amorphous Silica–Alumina Derived from Sugar Cane Bagasse Ash as an Ethylene Scavenger for Extending Shelf Life of Mango Fruits
Source: ACS Omega. 2024 Feb 2;9(6):6749–60. doi: 10.1021/acsomega.3c08119 (PMC10870304; doi:10.1021/acsomega.3c08119)
Supplement: Supplementary file 1 — ao3c08119_si_001.pdf [file ao3c08119_si_001.pdf]

Supporting Information for

Potassium Permanganate-Impregnated Amorphous Silica-Alumina Derived from Sugarcane Bagasse Ash as an Ethylene Scavenger for Extending Shelf-Life of Mango Fruits

Napassorn Chanka[1], Waleeporn Donphai[1], Metta Chareonpanich[1,2], Kajornsak Faungnawakij[3], Günther Rupprechter[4], Anusorn Seubsai[1,2]*

[1] Department of Chemical Engineering, Faculty of Engineering, Kasetsart University, Bangkok 10900, Thailand

[2] Center of Excellence on Petrochemical and Materials Technology, Kasetsart University, Bangkok 10900, Thailand

[3] National Nanotechnology Center (NANOTEC), National Science and Technology Development Agency (NSTDA), Pathum Thani 12120, Thailand

[4] Institute of Materials Chemistry, Technische Universität Wien, Getreidemarkt 9/BC, Vienna, 1060, Austria

***Corresponding author**: fengasn@ku.ac.th

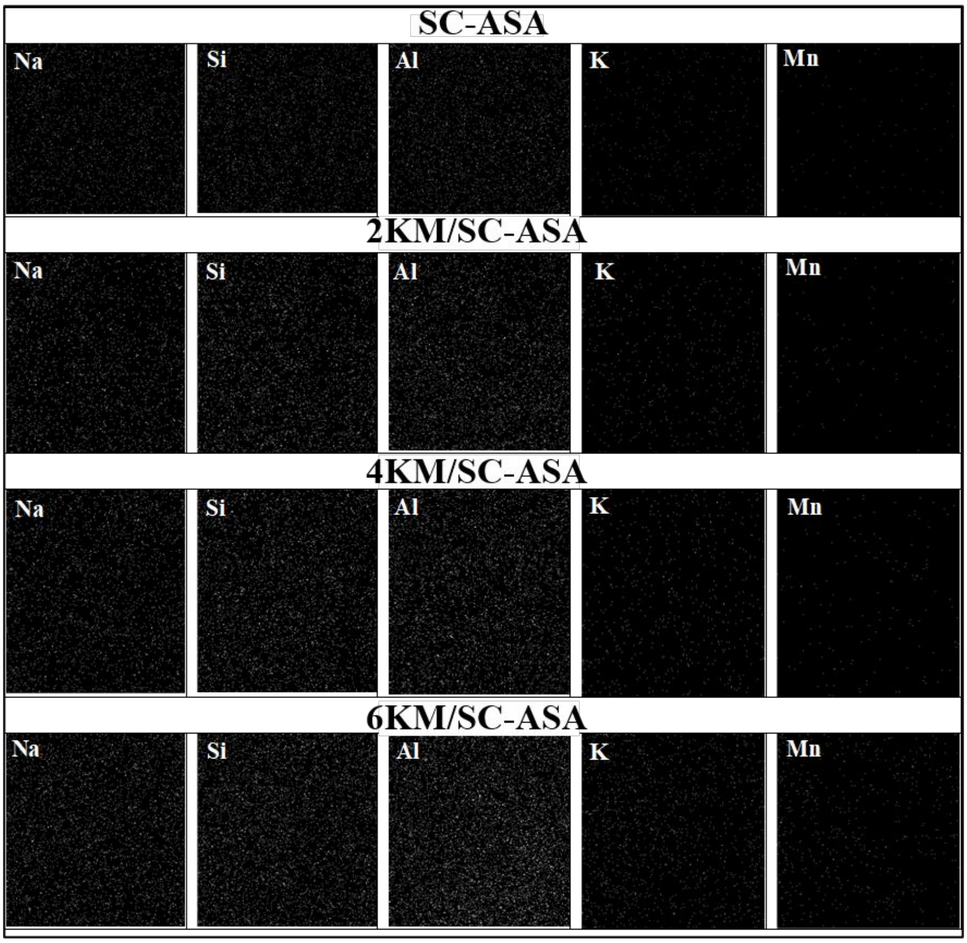

Figure S1. EDS-mapping of elements on the surface of all samples by SEM/EDS analysis.

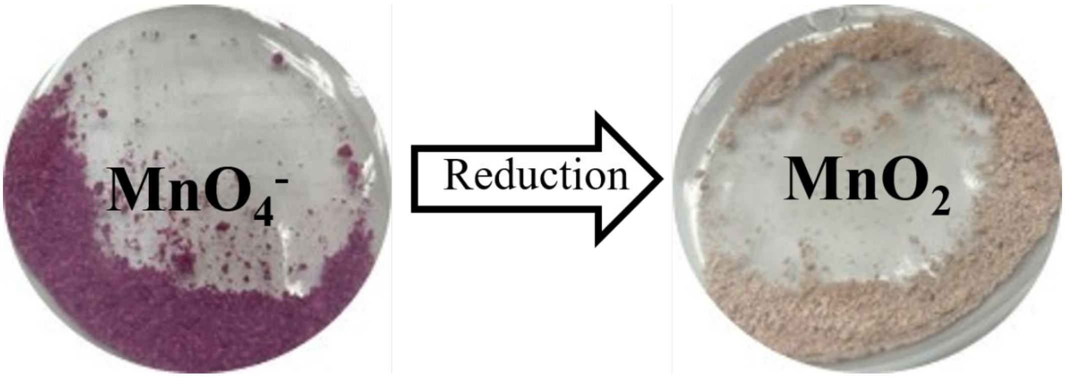

Figure S2. Image of the color changes in the C_2H_4 reduction of a $KMnO_4$-based SC-ASA sample.
